# Polyinfection secondary to exogenous lipoid pneumonia caused by aspiration of paraffin oil: a Case Report

**DOI:** 10.3389/fmed.2025.1555471

**Published:** 2025-08-01

**Authors:** Xiaoru Yan, Xun Chen, Yanan Zhu, Shu Chen, Xiangrui Meng, Jie Li, Yufeng Tang, Wei Zhang, Liang Yi, Yan Fu

**Affiliations:** Xiyuan Hospital of China Academy of Chinese Medical Sciences, Beijing, China

**Keywords:** exogenous lipoid pneumonia, paraffin oil aspiration, polymicrobial infections, corticosteroid therapy, bronchoalveolar lavage, SARS-CoV-2

## Abstract

**Background:**

Exogenous lipoid pneumonia is a rare condition caused by the aspiration of lipid-containing substances. Its diagnosis can be challenging due to non-specific respiratory symptoms and imaging findings. Complications, such as secondary infections, may exacerbate the clinical course, particularly in immunocompromised or elderly patients. No cases of ELP complicated by concurrent viral (SARS-CoV-2), bacterial, and fungal infections have been reported in recent literature.

**Case Presentation:**

We present the case of a 90-year-old female who developed ELP following the use of oral paraffin oil for ileus management. Initial symptoms included fever, cough, and respiratory distress, with rapidly worsening dyspnea. A chest CT revealed fat-density consolidations. Lipid droplets and lipid-laden macrophages were found in the bronchoalveolar lavage fluid. Her condition was further complicated by polymicrobial infections, including SARS-CoV-2, *Mycobacterium abscessus*, *Klebsiella pneumoniae*, and *Candida albicans*. A comprehensive treatment approach combining antibiotics, antifungals, antivirals, and corticosteroids resulted in significant clinical and radiological improvement over 1 week.

**Conclusion:**

This case underscores the importance of early recognition of ELP, particularly in high-risk populations, and highlights the need for a multidisciplinary approach to manage secondary infections. Systemic corticosteroids and tailored antimicrobial therapy proved effective in this critically ill patient.

## Background

Exogenous lipoid pneumonia is a condition caused by the aspiration of oily substances into the lungs. It leads to symptoms such as acute and chronic lung inflammation, local pulmonary fibrosis, and granuloma formation, all of which can impair gas exchange and, in severe cases, result in respiratory failure or death (1). While these pathological features are useful for diagnosing ELP, respiratory symptoms and imaging findings lack specificity. Additionally, due to its rarity, many clinicians are unfamiliar with ELP, leading to a high rate of misdiagnosis.

In ELP patients with rapid disease progression or poor response to treatment, clinicians should consider the possibility of secondary infections caused by other microorganisms, as these may complicate the clinical course and require additional interventions to manage effectively. Although there have been some reports of secondary infections in ELP, the pathogens involved are generally uncomplicated (2, 3). We hereby report a case of mixed infections following the diagnosis of ELP, including viral (SARS-CoV-2), bacterial, and fungal infections. Triple infections with bacteria, fungi, and viruses in ELP have been reported in children (4), while adult cases are extremely rare. To our knowledge, this is the first reported case of triple infection identified in a PubMed literature review spanning the last 5 years.

## Case presentation

A 90-year-old Chinese female with a significant medical history of ischemic stroke presented to the gastroenterology department following 3 weeks of altered mental status and recurrent vomiting. Initial diagnostic considerations included intestinal obstruction. She was administered oral paraffin oil (50 mL tid) for 3 days starting October 26, 2024, as part of the management. There were no significant respiratory symptoms until the night of October 29, when the patient developed fever (maximum temperature 37.6°C), coughing with a small amount of white sticky phlegm. A complete blood count was almost normal, but with elevated neutrophil percentage, and procalcitonin level was 0.224 ng/ml. She was preliminarily diagnosed with a pulmonary infection and started on intravenous etimicin sulfate 0.1 g qd. On November 1, the patient suddenly experienced chest tightness, dyspnea, and profuse sweating, unable to lie flat. Examination revealed a heart rate of 136 beats/min, blood pressure of 200/110 mmHg, widespread dry wheezing sound, and few moist rales in both lungs. Echocardiography indicated reduced left ventricular diastolic function, and she was treated with diuretics and bronchodilators.

**FIGURE 1:**
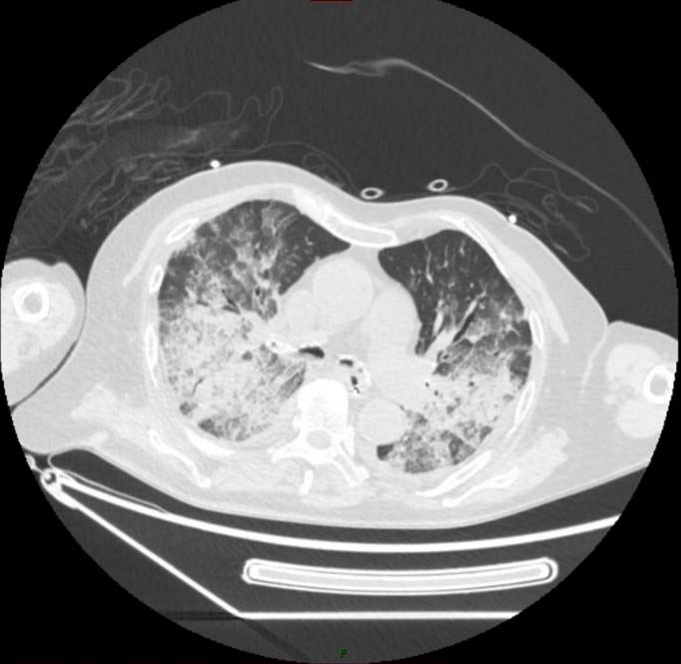
2024-11-4 chest CT shows multiple patchy and consolidated shadows in both lungs, with the CT value within the consolidated areas consistent with fat density.

By November 3, 2024, her respiratory distress markedly worsened, leading to coma, and she was subsequently transferred to the ICU for treatment. Lung sounds were coarse with evident rhonchi and rales. Laboratory tests showed significantly elevated infection markers (procalcitonin 5.480 ng/ml, CRP 158.54 mg/L), a remarkable rise in white blood cells and neutrophil percentage, and arterial blood gas analysis indicated metabolic acidosis and hypoxemia. A chest CT scan performed on November 4 revealed multiple patchy, confluent, and ill-defined infiltrates with areas of consolidation in both lungs. Notably, the consolidation areas showed fat density on CT, raising high suspicion for exogenous lipoid pneumonia (ELP) (Figure 1). Given the clinical findings, the patient underwent bronchoalveolar lavage (BAL) for diagnostic confirmation. On the morning of November 6, bronchoscopic examination revealed significant hyperemia and edema of the airway mucosa, with erythematous walls and numerous scattered petechial hemorrhages. Copious brownish-red secretions were noted in the left and right main bronchi, which were suctioned and collected for culture. The right middle lobe bronchus exhibited marked edema. 50 ml of bronchoalveolar lavage (BAL) fluid were obtained, with one aliquot submitted for pathological analysis and the other for metagenomics next-generation sequencing (mNGS) (Shengting Medical, utilizing MultiCapX coupled with a third-generation nanopore sequencing platform, covering the entire genome of bacteria, fungi, and viruses). In response, the patient was initiated on low-dose corticosteroid therapy with hydrocortisone (100 mg every 12 h) to alleviate edema and reduce inflammation. Concurrently, the patient was started on empirical antibiotic therapy with moxifloxacin and piperacillin/tazobactam, considering the possibility of a worsening pulmonary infection.

**FIGURE 2:**
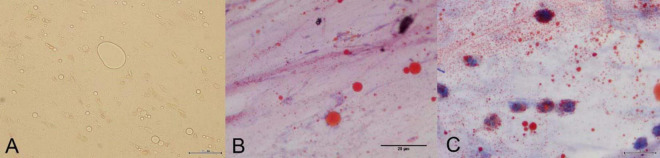
**(A)** Microscopic examination reveals fat droplets of varying sizes. **(B)** The bronchoalveolar lavage fluid shows positive Oil Red O staining. Microscopically, red-stained particles of varying sizes are observed. **(C)** Macrophages phagocytosing lipids are observed under the microscope.

**FIGURE 3:**
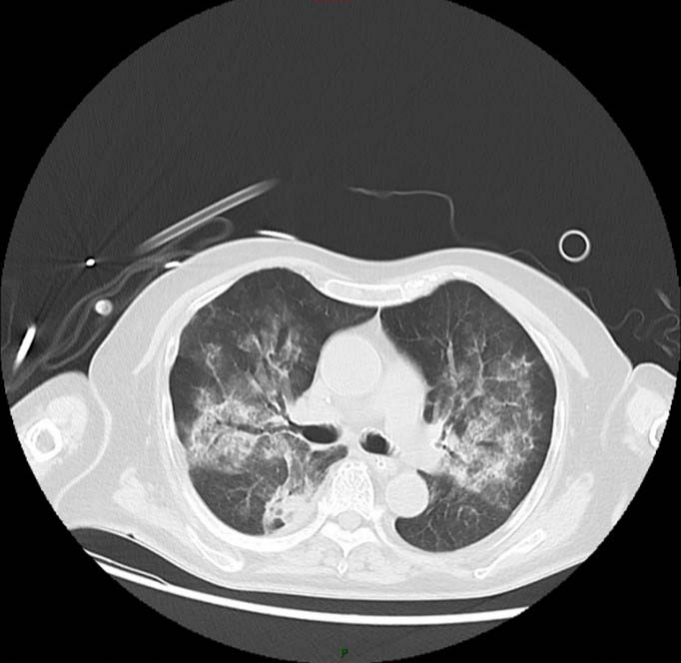
After 1 week of treatment, the patient’s chest CT shows significant improvement in the patchy shadows and consolidated areas in both lungs.

On November 7, the pathology report confirmed the presence of scattered fat droplets in the BAL fluid, which were positive for Oil Red O staining, confirming the diagnosis of exogenous lipoid pneumonia (Figure 2). Then mNGS results revealed a polymicrobial infection, including *Mycobacterium abscessus*, *Klebsiella pneumoniae*, *Pseudomonas aeruginosa*, *Acinetobacter baumannii*, *Enterococcus faecium*, *Candida albicans*, *cytomegalovirus (CMV), parvovirus B19, and SARS-CoV-2*. Sputum culture results indicated the presence of *Candida albicans, Acinetobacter baumannii*, and *Enterococcus faecium*, while BAL microscopy revealed a large number of hyphae. And the patient’s nucleic acid test for SARS-CoV-2 was positive. Given the multiple identified pathogens, the treatment regimen was adjusted to include a combination of piperacillin/tazobactam, levofloxacin, fosfomycin, ritonavir/nirmatrelvir, and liposomal amphotericin B for fungal coverage. Methylprednisolone was added to control potential cytokine storms in the setting of viral and bacterial co-infections. The patient’s clinical condition progressively improved with this combined therapy. A follow-up chest CT on November 11 showed marked improvement, with significant resolution of the patchy and consolidative opacities in both lungs (Figure 3).

## Discussion

The patient in this case presented with acute respiratory distress, progressive dyspnea, and hypoxemia, and was ultimately diagnosed with ELP. This rare pulmonary condition is typically caused by the inhalation of lipid-containing substances, such as mineral oil, which is commonly used in the management of chronic constipation in elderly patients. The high suspicion for ELP in this case was supported by imaging and laboratory findings, including characteristic lung CT findings, fat droplets and lipid-laden macrophages in the bronchoalveolar lavage (BAL). The diagnosis of ELP was further complicated by the presence of multiple concurrent infections, including viral (SARS-CoV-2), bacterial (*Klebsiella pneumoniae*, *Acinetobacter baumannii*, and *Enterococcus faecium*), and fungal (*Candida albicans*) infections.

Exogenous lipoid pneumonia primarily affects populations with aspiration risks, including elderly individuals, children, or patients with dysphagia. It is well-documented that aspiration of exogenous lipids triggers an inflammatory cascade leading to chronic granulomatous reactions, alveolar hemorrhage, and pulmonary fibrosis in severe cases (5). The patient’s history of dysphagia and prolonged use of mineral oil for constipation placed her at high risk. This aligns with findings in prior studies, where mineral oil aspiration has been associated with extensive lung consolidation and poor prognosis in geriatric populations with compromised immunityated with extensive lung consolidation and poor prognosis in geriatric populations with compromised immunity.

The diagnosis of exogenous lipoid pneumonia (ELP) is primarily based on chest CT findings, supplemented by relevant exposure history and bronchoalveolar lavage (BAL). Chest CT often reveals features such as ground-glass opacities (GGO), interlobular septal thickening, crazy paving, and bronchial dilatation. Areas of consolidation may show fat attenuation foci (<−30 Hu), which is a characteristic CT finding. We recommend routine chest CT screening for high-risk populations. BAL may demonstrate lipid-laden macrophages, which can exhibit large vacuoles and be positive for Oil Red O staining. Treatment focuses on discontinuing exposure to the lipid source. For more severe cases, systemic corticosteroid therapy (e.g., prednisolone, methylprednisolone) can be considered (6–10). The patient’s clinical response, as reflected by the improvement in inflammatory markers and the reduction in imaging abnormalities, confirms the potential benefits of corticosteroid therapy.

Therapeutic lung lavage might be an effective and safe therapy with long-term benefits for ELP (11). The whole lung lavage (WLL) is particularly useful in patients who fail to respond to conservative measures or systemic corticosteroid therapy (12). By physically removing lipid-laden material from the alveoli, WLL may improve lung function and oxygenation. However, this procedure carries inherent risks, especially in critically ill patients with poor cardiorespiratory reserve. In the present case, the risk-benefit analysis, considering the patient’s fragile condition and multiple infections, led to the decision to prioritize medical therapy over invasive interventions.

Most ELP cases secondary to single or mixed infections occur in children. A systematic review (4) showed that the predominant pathogens include *Mycobacterium fortuitum*, *M. fortuitum/chelonei*, *M. smegmatis*, and *M. abscessus* complex. Other reported infections encompass *Branhamella catarrhalis*; *Pseudomonas*, *Acinetobacter*, and *Klebsiella* spp.; respiratory viruses (e.g., adenovirus, human metapneumovirus, bocavirus, rhinovirus, parainfluenza, RSV A/B, coronavirus); various bacteria and fungi; *Chlamydia* (serologically confirmed); and peritoneal isolates such as *Enterococcus*, *Escherichia coli*, *Klebsiella*, coagulase-positive *Staphylococcus*, and *Clostridium*. Secondary infections in adult ELP are predominantly caused by bacteria or fungi. Bacterial pathogens include non-tuberculous mycobacteria (2, 3), *Acinetobacter baumannii* (9) and *Klebsiella pneumoniae* (8), while fungal pathogens are mostly *Candida* (8, 9). Relevant literature on this topic remains limited. Triple infections involving viruses, bacteria, and fungi are extremely rare. A search of PubMed literature over the past 5 years revealed that this case represents the first reported case of such multiple infections. Multiple infections are more common in immunocompromised populations such as children (4). The patient in this case had no history of definite immunodeficiency, and the multiple infections may be associated with advanced age (90 years), repeated hospitalizations, and recurrent aspiration due to swallowing dysfunction.

In recent years, metagenomic next-generation sequencing (mNGS) has been increasingly integrated into clinical pathogen detection workflows (13, 14). Distinguished by its enhanced efficiency, superior sensitivity, rapid turnaround time, and broad-spectrum coverage, mNGS enables simultaneous identification of bacteria, fungi, viruses, mycobacteria, parasites, and atypical pathogens (13, 15). A proof-of-concept study (16) has demonstrated the technical feasibility and clinical utility of metagenomic next-generation sequencing (mNGS) for diagnosing hospital-acquired pneumonia (HAP) and ventilator-associated pneumonia (VAP) in intensive care unit (ICU) settings. The findings showed strong concordance with culture-based diagnostics and enabled the identification of pathogens in pneumonia cases with initially undetermined etiology. Another study (17) has demonstrated that metagenomic next-generation sequencing (mNGS) provides clinical utility in guiding the management of severe hospital-acquired pneumonia (HAP). For this patient, we adopted a combined approach of sputum culture and bronchoalveolar lavage (BAL) mNGS for pathogen detection.

Sputum culture and mNGS revealed multiple pathogens, among which *Candida albicans* and *Klebsiella pneumoniae* are common colonizers of the upper respiratory tract, potentially aspirated into the lungs with paraffin oil due to swallowing dysfunction to form local infection foci. The infection of *Mycobacterium abscessus* may be associated with changes in the pulmonary microenvironment, and disinfection management loopholes of medical devices cannot be excluded. *Acinetobacter baumannii* and *Enterococcus faecium* are common drug-resistant bacteria in the ICU, and the patient was highly likely infected after transfer to the ICU. SARS-CoV-2 infection was considered cross-transmission during hospitalization. To this end, the patient was isolated in a single-room ward to prevent the transmission of multidrug-resistant bacteria and viruses. Antimicrobial therapy was tailored accordingly, including piperacillin-tazobactam, levofloxacin, caspofungin, and inhaled liposomal amphotericin B, in conjunction with antiviral agents nirmatrelvir/ritonavirnjunction. The multidisciplinary approach ensured comprehensive coverage of the infectious spectrum while closely monitoring inflammatory markers such as CRP and IL-6. Due to the partially similar imaging manifestations of ELP and pulmonary infections, it is challenging to differentiate between the two in a timely manner. Furthermore, ELP often leads to secondary infections due to compromised lung function. Therefore, the empirical use of antibiotics is essential (18).

Exogenous lipoid pneumonia typically has a benign prognosis when diagnosed early and treated appropriately. Once ELP is identified, immediate discontinuation of lipid substances can reduce intrapulmonary lipid deposition and avoid inflammatory cascade reactions. As reported in the literature, early administration of glucocorticoids (such as methylprednisolone) can significantly improve patients’ symptoms (6). In this case, the patient initiated glucocorticoid therapy immediately upon diagnosis, and the infection was observed to be controlled, indicating that early intervention yields better outcomes. ELP patients are predisposed to secondary infections due to alveolar lipid deposition disrupting the local immune barrier. Early diagnosis allows for close monitoring of infection markers and respiratory pathogen screening, enabling timely initiation of empirical anti-infective therapy to prevent single infections from progressing to mixed infections with multidrug-resistant bacteria (1).

However, delayed diagnosis, comorbid conditions, and severe infections may contribute to poor outcomes. This case underscores the multifactorial nature of ELP management, including early identification of lipid exposure, radiological confirmation, anti-inflammatory therapy, and aggressive management of secondary infections. Further studies are needed to establish optimal treatment protocols and clarify the role of interventions such as WLL in critically ill patients. Clinicians should maintain a high index of suspicion for ELP in patients presenting with unexplained respiratory symptoms and a history of oil ingestion.

## Data Availability

The original contributions presented in this study are included in this article/supplementary material, further inquiries can be directed to the corresponding author.
